# The Photoperiod Stress Response in *Arabidopsis thaliana* Depends on Auxin Acting as an Antagonist to the Protectant Cytokinin

**DOI:** 10.3390/ijms23062936

**Published:** 2022-03-08

**Authors:** Manuel Frank, Anne Cortleven, Aleš Pěnčík, Ondrej Novak, Thomas Schmülling

**Affiliations:** 1 Institute of Biology/Applied Genetics, Dahlem Centre of Plant Sciences (DCPS), Freie Universität Berlin, D-14195 Berlin, Germany; mfrank@mbg.au.dk (M.F.); anne.cortleven@fu-berlin.de (A.C.); 2 Laboratory of Growth Regulators, Faculty of Science, Palacký University & Institute of Experimental Botany, The Czech Academy of Sciences, Šlechtitelů 27, CZ-783 71 Olomouc, Czech Republic; alespencik@seznam.cz (A.P.); ondrej.novak@upol.cz (O.N.)

**Keywords:** *Arabidopsis thaliana*, abiotic stress, auxin, crosstalk, cytokinin, photoperiod stress

## Abstract

Fluctuating environmental conditions trigger adaptive responses in plants, which are regulated by phytohormones. During photoperiod stress caused by a prolongation of the light period, cytokinin (CK) has a protective function. Auxin often acts as an antagonist of CK in developmental processes and stress responses. Here, we investigated the regulation of the photoperiod stress response in *Arabidopsis thaliana* by auxin and its interaction with CK. Transcriptome analysis revealed an altered transcript abundance of numerous auxin metabolism and signaling genes after photoperiod stress treatment. The changes appeared earlier and were stronger in the photoperiod-stress-sensitive CK receptor mutant *arabidopsis histidine kinase 2* (*ahk2*),*3* compared to wild-type plants. The concentrations of indole-3-acetic acid (IAA), IAA-Glc and IAA-Asp increased in both genotypes, but the increases were more pronounced in *ahk2,3*. Genetic analysis revealed that the gain-of-function *YUCCA 1* (*YUC1*) mutant, *yuc1D*, displayed an increased photoperiod stress sensitivity. In contrast, a loss of the auxin receptors TRANSPORT-INHIBITOR-RESISTANT 1 (TIR1), AUXIN SIGNALING F-BOX 2 (AFB2) and AFB3 in wild-type and *ahk2,3* background caused a reduced photoperiod stress response. Overall, this study revealed that auxin promotes response to photoperiod stress antagonizing the protective CK.

## 1. Introduction

In plants, biotic and abiotic factors in the environment trigger adaptation responses which are regulated by a variety of signaling molecules. A new type of abiotic stress named photoperiod stress is caused by a prolongation of the photoperiod followed by a dark period [1,2]. It was first found in five-week-old short-day (SD) grown plants that were exposed once to a prolonged light period (PLP) of 32 h [3,4]. A shorter PLP of 4 h is also sufficient to induce a stress response [3,5]. Photoperiod-stress-exposed plants respond by an altered expression of photoperiod stress marker genes (e.g., *ZAT12*, *BAP1*, *CAB2*) about five hours after PLP treatment and by an oxidative burst due to the accumulation of apoplastic ROS during the night following the PLP treatment [3,5]. Upon strong stress treatments, leaves display a reduced photosynthetic efficiency and an increased percentage of lesion formation, which may ultimately lead to programmed cell death.

Recent work has shown that cytokinin (CK) and in particular root-derived *trans*-zeatin (*t*Z) have a protective function against photoperiod stress [6]. Reverse genetic approaches identified the receptors ARABIDOPSIS HISTIDINE KINASE 2 (AHK2) and AHK3, phosphotransferases ARABIDOPSIS HISTIDINE PHOSPHOTRANSFER PROTEIN 2 (AHP2), AHP3 and AHP5 and transcription factors ARABIDOPSIS RESPONSE REGULATOR 2 (ARR2), ARR10 and ARR12 as the main CK signaling components during the stress response [3,6]. Together, these results add to the emerging role of CK in a variety of stress responses [7,8,9].

Auxins are another well-studied class of phytohormones crucial for many developmental and stress-related processes [10]. IAA is the most important auxin in *Arabidopsis* and can be formed tryptophan-dependently or -independently [11]. The tryptophan-dependent indole-3-pyruvic acid (IPA) pathway is the main contributor to IAA formation in *Arabidopsis* [12,13]. During the initial step, TRYPTOPHAN AMINOTRANSFERASE OF ARABIDOPSIS 1 (TAA1) and TRYPTOPHAN-AMINOTRANSFERASE-RELATED (TAR) proteins convert tryptophan to IPA [14], which is further converted to bioactive free IAA by YUC proteins.

IAA signals intracellularly through TIR1/AFB receptors [15,16,17], AUX/IAA repressor proteins and AUXIN RESPONSE FACTOR (ARF) transcription factors [18,19]. Perception of IAA by TIR1/AFB receptors results in an SKP1-CULLIN-F-BOX (SCF)-TIR1/AFB complex formation that disrupts Aux/IAA function by mediating their ubiquitination and thus their degradation [15,20]. Consequently, the Aux/IAA repression of ARFs is relieved, and they become active. Structurally and functionally, ARFs can be divided in class A ARFs, whose members act mostly as transcriptional activators, and class B and C ARFs which act as repressors [21,22,23].

The most common irreversible IAA inactivation processes in *Arabidopsis* are the oxidation of IAA by two DIOXYGENASE FOR AUXIN OXIDATION (DAO) homologs [24,25] resulting in 2-oxoindole-3-acetic acid (oxIAA) and the conjugation of IAA with aspartate (Asp) or glutamate (Glu) by subfamily II of GRETCHEN HAGEN 3 (GH3) proteins [26,27] leading to the formation of IAA aspartic acid (IAA-Asp) and IAA glutamic acid (IAA-Glu). Both inactivation branches act redundantly [24]. GH3s also catalyze the reversible formation of other IAA-amino acid conjugates such as IAA-alanine (IAA-Ala) and IAA-leucine (IAA-Leu). Hydrolysis of these IAA conjugates is catalyzed by IAA-ALANINE-RESISTANT 3 (IAR3), IAA-LEUCINE-RESISTANT 1 (ILR1) and IAA-LEUCINE-RESISTANT-LIKE (ILL) proteins [28,29].

In numerous developmental processes such as embryogenesis or root development [30,31] but also in stress-related processes [10], auxin and CK signaling pathways interact by reciprocal regulation of their metabolism, signaling and transport [32]. Whether auxin is involved in the photoperiod stress response and if it interacts with CK in this context is not known.

Here, we report on the role of auxin in photoperiod stress and its interaction with CK in regulating the response to photoperiod stress. Upon exposure to a PLP, an altered transcript abundance of numerous auxin genes was observed in wild-type and *ahk2,3* leaves. Both genotypes accumulated IAA and IAA conjugates in response to photoperiod stress. This increased auxin status resulted in an enhanced photoperiod stress response, whereas an impairment in auxin perception reduced the stress sensitivity in both wild type and *ahk2,3*. Overall, our study establishes auxin as an important player in regulating the sensitivity to extended light periods and adds photoperiod stress to the list of processes in which CK and auxin are interacting key regulators.

## 2. Results

### 2.1. Auxin-Related Genes Are Differentially Regulated in Response to Photoperiod Stress

**Figure 1:**
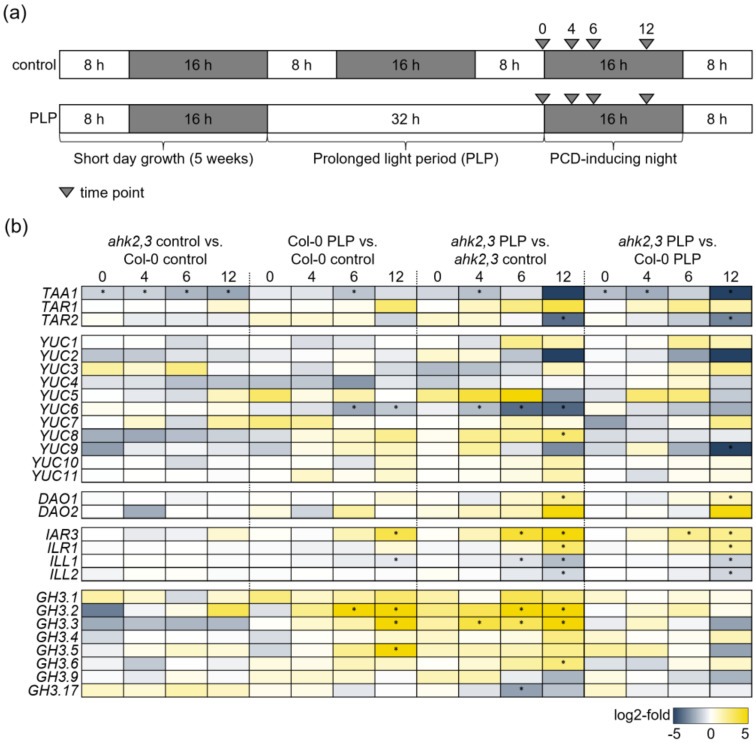
Relative expression of auxin biosynthesis and metabolism genes in response to photoperiod stress in wild type and *ahk2,3*. (**a**) Schematic overview of photoperiod stress treatment and sampling time points for gene expression analysis (0, 4, 6 and 12 h after PLP treatment; gray arrows). (**b**) Relative expression levels of auxin biosynthesis and metabolism genes *TRYPTOPHAN AMINOTRANSFERASE OF ARABIDOPSIS* (*TAA1*), *TAA-RELATED* (*TAR*), *YUCCA* (*YUC*), *DIOXYGENASE FOR AUXIN OXIDATION* (*DAO*), *IAA-ALANINE-RESISTANT 3* (*IAR3*), *IAA-LEUCINE-RESISTANT 1* (*ILR1*), *IAA-LEUCINE-RESISTANT-LIKE* (*ILL*) and *GRETCHEN HAGEN 3* (*GH3*) 0, 4, 6 and 12 h after the PLP treatment compared to respective control plants. Stars indicate a significant difference between the indicated genotypes/conditions (*p* ≤ 0.05; *n* = 3; log_2_-fold values are depicted in Supplementary Table S1). Data were extracted from [33].

**Figure 2:**
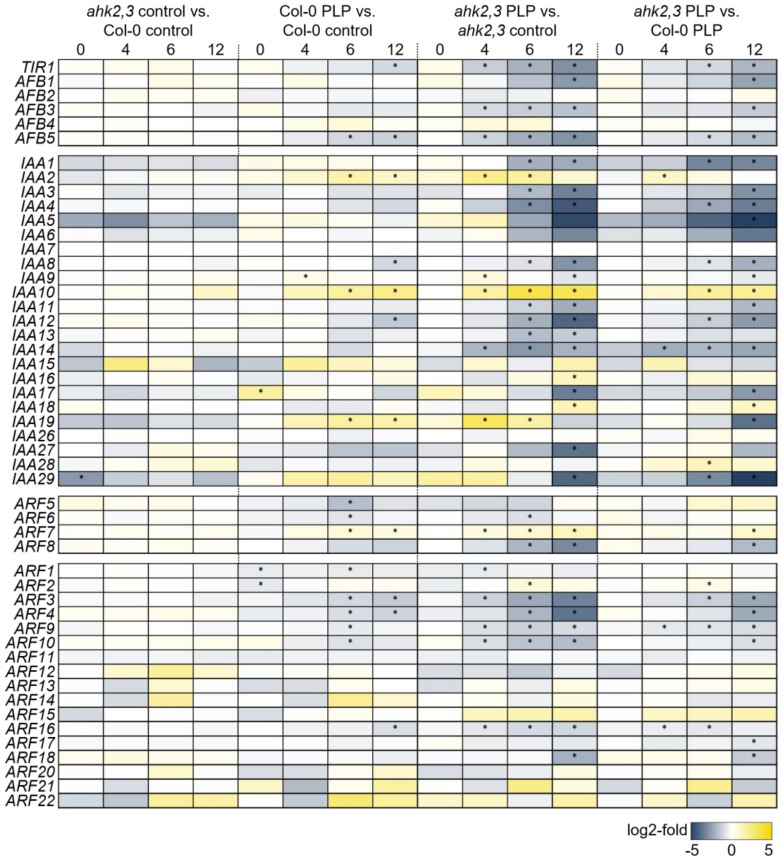
Relative expression of auxin signaling genes in response to photoperiod stress in wild type and *ahk2,3*. Depicted are the relative expression of *TRANSPORT-INHIBITOR-RESISTANT 1* (*TIR1*)/*AUXIN SIGNALING F-BOX* (AFB), *Aux*/*IAA* and *AUXIN RESPONSE FACTORs* (*ARFs*) (class A ARFs, transcriptional activators, first group; class B and C, transcriptional repressors, second group) 0, 4, 6 and 12 h after the PLP compared to respective control plants. Stars indicate a significant difference between the indicated genotypes/conditions (*p* ≤ 0.05; *n* = 3; log_2_-fold values are depicted in Supplementary Table S2). Data were extracted from [33].

To investigate the possible involvement of auxin in the photoperiod stress response, we first analyzed the transcript abundance of genes involved in auxin synthesis, metabolism and signaling after photoperiod stress. The changes of transcript abundance were compared in leaves of stressed (PLP) and non-stressed (control) wild-type and *ahk2,3* plants 0, 4, 6 and 12 h after PLP treatment using data from RNA-seq analysis (Cortleven et al. [33]; Figure 1 and Figure 2; Supplementary Tables S1 and S2). The changes in transcript abundance in response to photoperiod stress were confirmed for several genes by qRT-PCR (Supplementary Figure S1).

Under control conditions, the abundance of all transcripts of auxin-related genes was similar in wild type and *ahk2,3*, except for *TAA1*, which showed a significantly lower expression level in *ahk2,3* compared to wild type at all time points (Figure 1b). Photoperiod stress treatment caused strong changes in the expression of several auxin genes, particularly at later time points and in the *ahk2,3* mutant. In wild type, the abundance of *IAR3*, *GH3.2*, *GH3.3* and *GH3.5* was increased more than ten-fold 12 h after the PLP treatment while *YUC6* and *ILL1* were 2.7-fold and 1.5-fold less abundant compared to control plants. A similar regulation was observed in PLP-treated *ahk2,3* plants versus control plants, but the changes occurred earlier than in wild type (at 4 h and 6 h). In addition, *YUC8*, *DAO1* and *ILR1* were more abundant, and *TAR2* and *ILL2* were 14-fold and two-fold less abundant in *ahk2,3* PLP plants 12 h after the PLP treatment. The earlier and stronger changes of the transcript abundance of photoperiod-stress-responsive auxin genes in *ahk2,3* are in line with the recently described strong transcriptional responses of *ahk2,3* to photoperiod stress indicating an increased sensitivity compared to wild type [33].

The changes in the abundance of the transcripts of auxin signaling genes after PLP treatment followed a similar pattern as it was observed for auxin synthesis and metabolism genes (Figure 2). In wild-type PLP-treated plants, *TIR1* and *AFB5* transcripts were ca. two-fold less abundant compared to control plants 12 h after the PLP as were *IAA8*, *IAA12* and several *ARF* gene transcripts. *IAA2*, *IAA10* and *IAA19* were more abundant just like *ARF7*. In comparison, the abundance of these gene transcripts was more strongly changed in *ahk2,3* (PLP 12 h) compared to the respective control plants. For example, in wild-type, the expression of *AFB5* was decreased two-fold at the 12 h time point while it was decreased eight-fold in *ahk2,3*, where the decrease also started significantly earlier than in wild-type. Transcriptional changes of these genes were apparent in *ahk2,3* already 4 h and 6 h after the PLP, but the difference was strongest 12 h after the PLP. In addition, several additional *AFB*, *IAA* and *ARF* transcripts that were non-responsive in wild type displayed an altered abundance in *ahk2,3* after PLP treatment (Figure 2).

Summing up, the alterations in the abundance of auxin synthesis, metabolism and signaling gene transcripts in response to photoperiod stress were more pronounced in photoperiod-stress-sensitive *ahk2,3* plants compared to wild type. This provided first indications for the functional relevance of auxin in the context of photoperiod stress.

### 2.2. Photoperiod Stress Treatment Increases the Concentration of Free IAA in Wild-Type and ahk2,3 Plants

**Figure 3:**
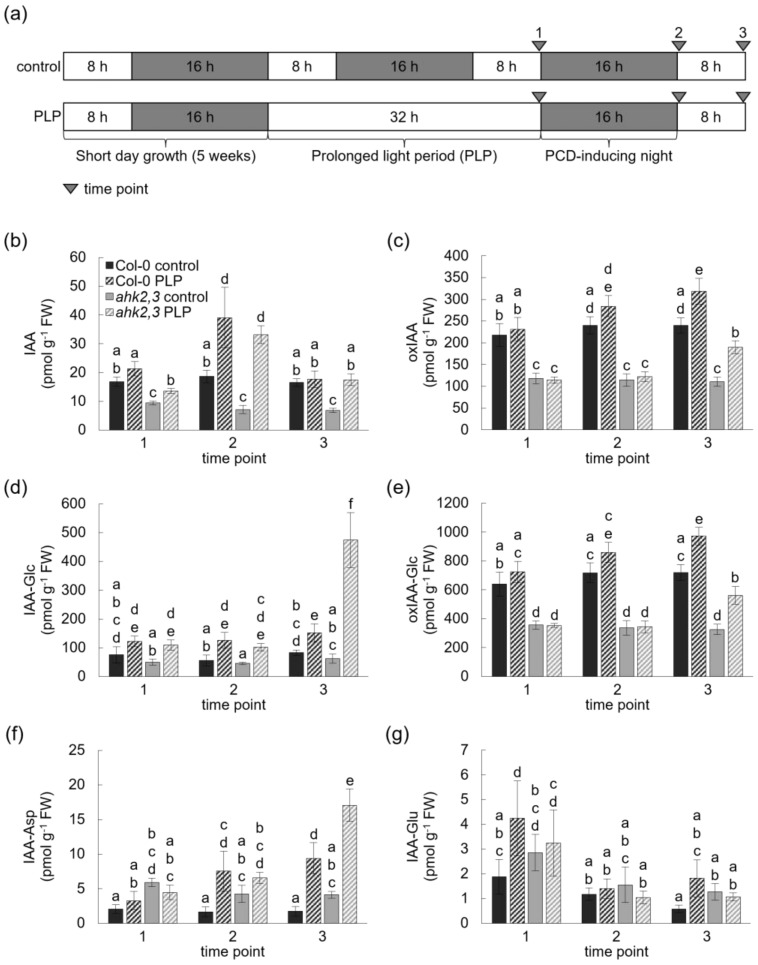
Photoperiod stress causes an increased concentration of IAA, IAA-Glc and IAA-Asp in wild-type and *ahk2,3* plants. (**a**) Schematic overview of sampling time points (arrowheads) for IAA measurements. Five-week-old wild-type plants were either cultivated under SD conditions (control) or were exposed to a prolonged light period of 32 h (PLP). (B–G) Concentration of free IAA (**b**), oxIAA (**c**), IAA-Glc (**d**), oxIAA-Glc (**e**), IAA-Asp (**f**) and IAA-Glu (**g**) in control samples and PLP samples at the time points depicted in (**a**). Letters indicate significantly different statistical groups (*p* ≤ 0.05; two-way ANOVA; *n* ≥ 3). Error bars indicate SD. IAA, indole-3-acetic acid; oxIAA, 2-oxoindole-3-acetic acid; IAA-Glc, IAA-glucose; IAA-Asp, IAA-aspartic acid; IAA-Glu, IAA-glutamic acid. Exact values are depicted in Supplementary Table S3.

As numerous genes involved in auxin biosynthesis and metabolism were differentially expressed particularly in PLP-treated *ahk2,3* plants, we further investigated the possible involvement of auxin in the response to photoperiod stress by measuring the concentrations of bioactive free IAA and several of its inactive derivatives after PLP treatment, after the end of the following night and the end of the next light period (Figure 3; Supplementary Table S3). Under control conditions, essentially no significant changes of free IAA and its metabolites were noted over time in wild type and *ahk2,3*. However, consistent with the low abundance of *TAA1* in *ahk2,3* (Figure 1b), the concentration of free IAA was reduced by 50% in *ahk2,3* control plants compared to wild type. The concentrations of oxIAA, IAA-Glc and oxIAA-Glc were also strongly reduced in *ahk2,3* (Figure 3b–e). In contrast, the concentrations of IAA-Asp and IAA-Glu were increased (Figure 3f,g). At the end of the PLP treatment (time point 1), no differences in the concentration of IAA and its derivatives were detected in wild-type PLP-treated plants compared to respective control plants, except for an increase in IAA-Glu (Figure 3b–g). In contrast, at that time point, *ahk2,3* plants displayed a ca. 40% increase in free IAA and a more than doubled IAA-Glc concentration compared to untreated controls (Figure 3b,d). After the night following the PLP treatment (time point 2), the IAA concentration increased in both genotypes compared to the respective controls (Figure 3b). The relative increase was about two-fold in wild type and over four-fold in *ahk2,3*; nevertheless, the final concentration was still lower in the latter. At the end of the following day (time point 3—after PLP), the IAA concentration was lowered again in both genotypes, reaching the original level in wild type and still being elevated in *ahk2,3*. At that time point, the concentrations of IAA-Glc and IAA-Asp differed strongly between control and PLP plants. There was a 1.8-fold and 7.5-fold increase in IAA-Glc and a 5.5-fold and 4.2-fold increase in IAA-Asp concentration in PLP-treated wild type and *ahk2,3*, respectively (Figure 3e,f).

Overall, the concentrations of free IAA and its inactive metabolites IAA-Glc and IAA-Asp increased in response to a PLP treatment followed by darkness. When comparing control and PLP-treated plants of the same genotype, the relative increase of IAA and its metabolites oxIAA, IAA-Glc and oxIAA-Glc was higher in *ahk2,3* compared to wild type. The stronger response of *ahk2,3* to photoperiod stress provided another indication for an involvement of auxin in the response to photoperiod stress.

### 2.3. Plants with an Impaired Auxin Perception Are Less Sensitive to Photoperiod Stress

**Figure 4:**
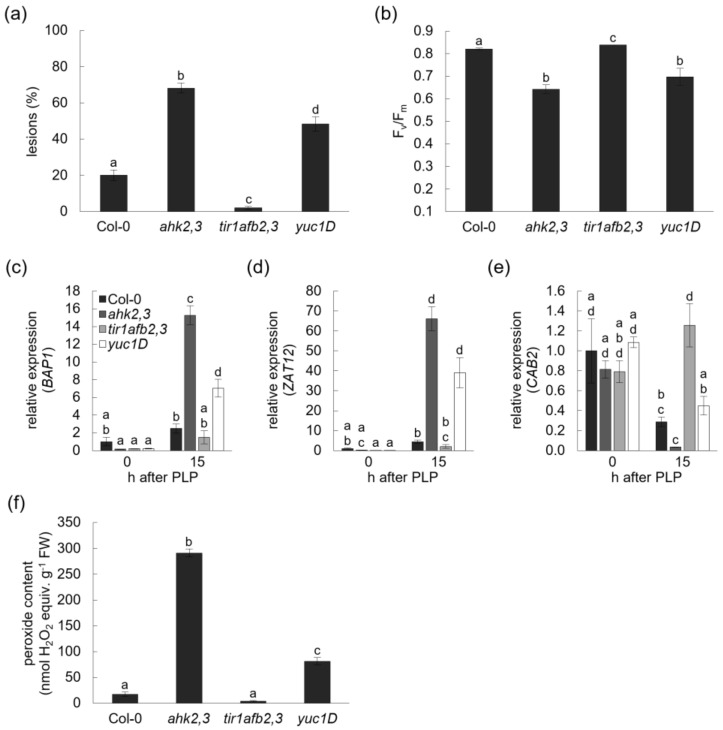
The auxin status influences the response to photoperiod stress. (**a**) Lesion formation of leaves in five-week-old Col-0, *ahk2,3*, *tir1afb2,3* and *yuc1D* plants the day after the photoperiod stress treatment. (**b**) Efficiency of photosystem II (F_v_/F_m_) of leaves the day after the photoperiod stress treatment (one-way ANOVA; *p* ≤ 0.05; *n* = 15). Transcript abundance of stress marker genes *BAP1* (**c**), *ZAT12* (**d**) and *CAB2* (**e**) 0 and 15 h after the PLP treatment compared to respective control plants. The expression level of wild type at the end of the PLP treatment (0 h) was set to 1 (one-way ANOVA; *p* ≤ 0.05; *n* ≥ 3). (**f**) Peroxide content expressed as nmol H_2_O_2_ equivalents g^−1^ FW in leaves 15 h after PLP treatment (one-way ANOVA; *p* ≤ 0.05; *n* = 4). Letters indicate significantly different statistical groups. Error bars indicate SE. Pictures of representative plants exposed to a 24 h prolongation of the light period are shown in Supplementary Figure S4a.

Next, plants with an altered auxin status were exposed to photoperiod stress. Analysis of lower- and higher-order auxin receptor mutants revealed that TIR1, AFB2 and AFB3 positively regulated the sensitivity to photoperiod stress in a redundant manner with *tir1afb2,3* plants being almost completely photoperiod-stress-insensitive (Figure 4 and Figure S2). In response to photoperiod stress, *tir1afb2,3* formed almost no lesions (Figure 4a), displayed no lowered F_v_/F_m_ (Figure 4b), showed only a low induction of stress response genes (Figure 4c,d) or even no stress response as in the case of *CAB2* (Figure 4e) and responded with a very low ROS formation (Figure 4f). In contrast, *yuc1D* plants, which have an increased auxin concentration, were more sensitive than wild type in terms of lesion formation but less sensitive than *ahk2,3* plants (Figure 4a). This response pattern, more sensitive than wild type but less sensitive than *ahk2,3*, was also seen in *yuc1D* plants for the stress marker genes and the formation of ROS (Figure 4c–f).

To sum up, plants with reduced auxin perception were more resistant to photoperiod stress while plants with an elevated auxin content were more sensitive. These results provide clear genetic evidence for a role of auxin in the response to photoperiod stress.

### 2.4. Impairment of Auxin Perception Reduces the Photoperiod Stress Response of the CK Receptor Mutant ahk2,3

**Figure 5:**
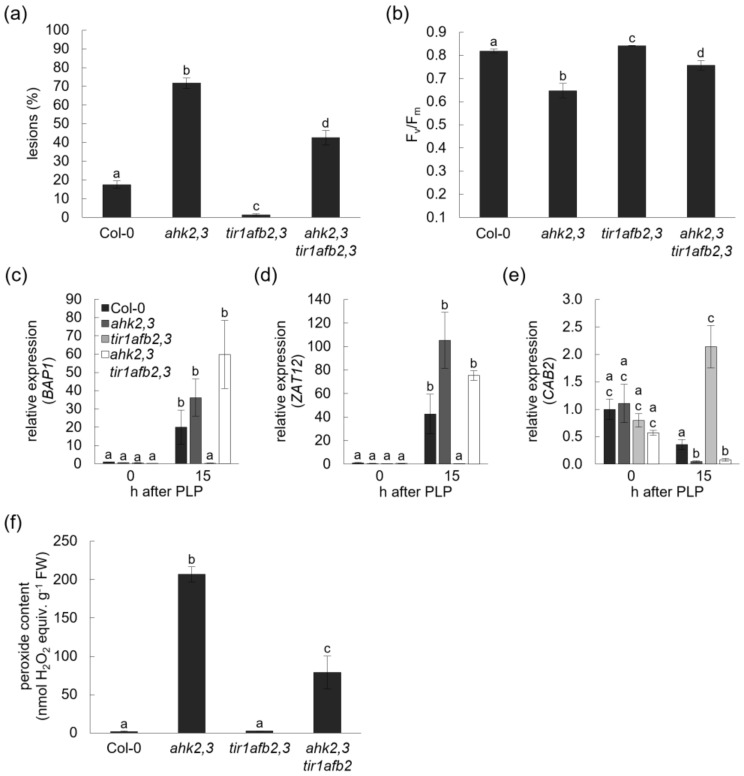
An impairment of auxin perception decreases the sensitivity to photoperiod stress. (**a**) Lesion formation of leaves in five-week-old Col-0, *ahk2,3*, *tir1afb2,3* and *ahk2,3 tir1afb2,3* plants the day after the photoperiod stress treatment (one-way ANOVA; *p* ≤ 0.05; *n* ≥ 12). (**b**) Efficiency of photosystem II (F_v_/F_m_) of leaves the day after photoperiod stress treatment (paired Wilcoxon test, FDR corrected via Benjamini–Hochberg method; *p* ≤ 0.05; *n* ≥ 13). Transcript abundance of *BAP1* (**c**), *ZAT12* (**d**) and *CAB2* (**e**), 0 and 15 h after the PLP treatment compared to respective control plants. The expression level of wild type at the end of the PLP treatment (0 h) was set to 1 (one-way ANOVA; *p* ≤ 0.05; *n* ≥ 3). (**f**) Peroxide content expressed as nmol H_2_O_2_ equivalents g^−1^ FW in Col-0, *ahk2,3*, *tir1afb2,3* and *ahk2,3 tir1afb2* leaves 15 h after PLP treatment (one-way ANOVA; *p* ≤ 0.05; *n* = 4). Letters indicate significantly different statistical groups. Pictures of representative plants exposed to a 24 h prolongation of the light period are shown in Supplementary Figure S4b.

As impairment in auxin perception caused a reduction in photoperiod stress sensitivity and plants with an impaired CK perception were more sensitive to photoperiod stress, a genetic interaction of both hormones was tested by generating *ahk2,3 tir1afb2,3* quintuple mutants and exposing these to photoperiod stress. The quintuple mutant showed for several parameters an intermediate phenotype, with a weaker stress phenotype than *ahk2,3* but a stronger stress phenotype than wild type or the *tir1afb2,3* mutant (Figure 5).

This was particularly clear for lesion formation, F_v_/F_m_, the response of *ZAT12* and ROS formation (Figure 5a,b,d,f). Notably, the strong induction of *BAP1* and the reduction in *CAB2* expression of stress-treated *ahk2,3* mutants were not rescued by introgression of auxin receptor mutations (Figure 5c,e).

Taken together, an impairment of auxin perception in *ahk2,3* reduced the photoperiod stress sensitivity demonstrating the antagonistic functions of auxin and CK in this context. CK might also act downstream of auxin for the transcriptional regulation of some photoperiod-stress-sensitive genes (*BAP1*, *CAB2*).

## 3. Discussion

Our study indicates that the auxin status of *Arabidopsis* plants is relevant for their response to photoperiod stress. Analyses investigating the transcriptomic responses to photoperiod stress revealed mostly minor changes in the abundance of gene transcripts encoding enzymes of auxin biosynthesis and metabolism in wild-type leaves, whereas the expression of these genes was more affected in CK-deficient *ahk2,3* (Figure 1 and Figure 2). Strikingly, the auxin biosynthesis gene *TAA1* was the only gene significantly less abundant in *ahk2,3* compared to wild type at all time points. TAA1 is crucial for the formation of IAA as it catalyzes the formation of its precursor IPA from Trp [14]. Its expression is promoted by two transcription factors, ARR1 and ARR12, known to mediate CK signals, which bind to the *TAA1* promoter [34], presenting a prime example of direct regulation of auxin metabolism by CK. It is conceivable that ARR1 and ARR12 are less active in CK receptor mutants, which might be the cause for a reduced auxin synthesis in *ahk2,3*.

Other transcriptional changes of auxin-related genes induced by photoperiod stress may affect the stress response. Several *GH3* gene transcripts were more abundant upon PLP treatment in both genotypes. The constitutive overexpression of these genes, *GH3.5* (*wes1-D* mutant), causes an IAA deficiency phenotype mainly by the synthesis of IAA-Asp and also increases the resistance to cold, drought and heat stress, while the *gh3.5* mutant has increased IAA levels and is less stress-resistant [35]. A decreased IAA concentration due to a gain-of-function mutation of *GH3.13* improved the resistance of rice to drought [36]. Other examples of regulation of *GH3* genes by CK have been reported for the regulation of root growth [37]. It is well established that CK and auxin regulate each other’s synthesis pathways and thus their hormone levels [34,38,39,40,41]. In addition, the stronger regulation of *GH3* genes in the CK-deficient *ahk2,3* adds a facet to this picture.

Apart from influencing auxin synthesis and metabolism genes, RNA-seq results show that with a developing photoperiod stress syndrome, the expression of genes involved in auxin signaling including receptors, activators and repressors was mostly decreased in wild type and even more strongly in photoperiod-stress-sensitive *ahk2,3* (Figure 3). The strong deregulation of these genes in *ahk2,3* agrees with previous studies indicating that *t*Z-dependent signaling is crucial to balance auxin synthesis by regulating *IAA3/SHORT HYPOCOTYL 2* (*SHY2*) and *IAA17/AXILLARY ROOT 3* (*AXR3*) expression [40]. In the context of photoperiod stress, it could be that one of the protective functions of CK, which increases after PLP treatment in wild-type plants [6], is to balance the increased IAA synthesis upon stress.

The auxin measurements conducted in this study support this idea. IAA, oxIAA and oxIAA-Glc concentrations were about 50% lower in *ahk2,3* control plants compared to wild-type controls (Figure 3b,c,e). This is in accordance with previous findings of a reduced IAA concentration in *Arabidopsis* plants with a decreased CK content [42,43]. As IAA oxidation is the main IAA inactivation pathway in *Arabidopsis* [24,25], the reduced oxIAA and oxIAA-Glc concentrations might reflect the decreased IAA concentration/synthesis in *ahk2,3* plants. After exposure to a PLP followed by darkness, the concentration of IAA and its inactivated derivatives oxIAA, IAA-Glc and oxIAA-Glc increased in both wild type and *ahk2,3*, but the relative increase was higher in the latter at the later time point (Figure 3b–e). The strong increase of these inactive derivatives in *ahk2,3* could hint at a homeostatic mechanism to prevent the accumulation of free IAA. One part of this mechanism could also be based on the substantial influence that CK has on auxin transport in the root and during de novo organogenesis [44,45,46,47].

Mutant analyses revealed that an increasing impairment in auxin perception by the mutation of *TIR/AFB* genes resulted in a decreased photoperiod stress sensitivity in terms of lesion formation, partially in marker gene expression and peroxide content. In contrast, an increase in the concentration of endogenous IAA in *yuc1D* increased the sensitivity to photoperiod stress (Figure 4 and Figure S2). This is congruent with studies reporting that an increased auxin status is associated with a decreased stress resistance to cold, drought and salt [35]. A decreased auxin status by either loss of *TIR/AFB* or by a constitutive overexpression of *GH3.5* (*wes1-D* mutant) enhanced the resistance to these stresses [35,48,49]. Other studies suggest that either auxin supplementation or an increase in endogenous IAA concentration by *YUC* overexpression improves the resistance to drought stress [50,51]. These opposing results indicate that balancing auxin concentration and auxin signaling is of crucial importance for the appropriate response to stress, but the outcome might be context-dependent. The decreased photoperiod stress symptoms of *ahk2,3 tir1afb2,3* compared to *ahk2,3* indicate hormonal crosstalk. Therefore, the increased sensitivity of plants with a reduced CK status might at least partly be due to an imbalance in the CK-auxin homeostasis (Figure 5), which is supported by the changes in auxin metabolite concentrations and altered transcript levels of auxin-related genes.

What might be the mechanism behind a potential toxicity of IAA in the context of photoperiod stress? Several studies suggest that auxin induces the formation of ROS during developmental processes such as the gravitropic response of roots [52], lateral root and root hair development [53,54], cell wall loosening [55] and quiescent center formation [56]. Moreover, auxin induces the formation of H_2_O_2_ during salt stress [48] acting through NADPH oxidases RESPIRATORY BURST OXIDASE HOMOLOGs (RBOHs), the content of ascorbic acid and the activity of superoxide dismutases (SODs), GLUTATHIONE REDUCTASEs (GRs) and CATALASEs (CATs) [51,53,57]. Together with the results of this study, one interpretation could be that the disproportional increase in IAA might induce oxidative stress ultimately resulting in the photoperiod stress syndrome after exposure to a PLP. Resistant plants therefore would be able to respond to the PLP by increasing IAA inactivation or by reducing the auxin signaling output ultimately reducing oxidative stress and thus the severity of stress symptoms.

**Figure 6:**
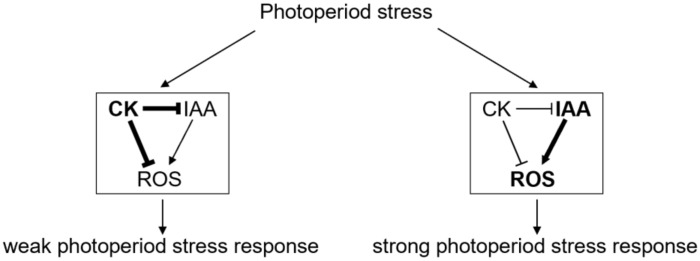
Model of the function and interaction of CK and auxin in the response to photoperiod stress. In leaves showing a weak photoperiod stress response (left side), CK accumulates (bold letters) and inhibits auxin (IAA) and ROS accumulation (thick black arrows). In leaves showing a strong photoperiod stress response (e.g., CK signaling or biosynthesis mutants; right side), CK does not sufficiently repress IAA synthesis or signaling resulting in its accumulation and enhanced signaling (bold letters). IAA ultimately induces ROS formation (bold letters and thick arrow).

In contrast to auxin, CK is associated with a decrease in ROS formation [9]. It acts as a negative regulator of ROS formation after high light stress [58], counteracts oxidative stress [59], balances ROS production during meristem development [60] and transcriptionally regulates *PEROXIDASE 33* (*PRX33*) and *PRX34* via ARR2 [61]. In response to photoperiod stress, CK signals through its receptors AHK2 and AHK3 to negatively regulate the formation of apoplastic ROS and *PRX4* and *PRX71* expression [5] and to induce the transcription of various other oxidative-stress-related genes [33]. Considering all of this, we hypothesize that CK acts as a protectant against photoperiod stress by either acting directly on the auxin status and/or by antagonizing auxin action by reducing ROS formation (Figure 6). Future studies investigating the transcriptional and metabolic CK status as well as the antioxidant system in auxin mutants during and after being exposed to a PLP will reveal more precisely how CK and auxin interact to regulate the photoperiod stress syndrome.

## 4. Materials and Methods

### 4.1. Plant Material and Growth Conditions

The Columbia-0 (Col-0) ecotype of *Arabidopsis thaliana* was used as the wild type. The following mutant and transgenic *Arabidopsis* plants were used in this study: *ahk2-5,3-7* [62], *yuc1D* [63,64] (obtained from Christine Beveridge) and *tir1-1afb2-3,3-4* [16,17] (obtained from the European Arabidopsis Stock Centre (NASC, Nottingham, UK; http://arabidopsis.info/, last accessed on 5 March 2022)). The *ahk2,3tir1afb2,3* quintuple mutant was generated by genetic crossing, and the genotypes were confirmed by PCR analysis. *Arabidopsis* plants were grown on soil in a growth chamber under SD conditions (8 h light/16 h dark) as described in Nitschke et al. [3].

### 4.2. Photoperiod Stress Treatment and Harvest of Leaf Material

A detailed description of the stress treatment performed and of the harvesting conditions can be found in Frank et al. [6]. A schematic overview of the used experimental conditions is shown in Figure 1a. Briefly, SD-grown five-week-old plants were exposed to a light period of 32 h (prolonged light period, PLP) followed by an SD. Control plants remained under SD conditions. For phenotypical and molecular analyses, leaves from stress-treated plants of the same developmental stage (leaves 8–12) were chosen. Harvest during the dark period was performed in green light.

### 4.3. Chlorophyll Fluorometry, Analysis of Cell Death Progression and ROS Measurement

Chlorophyll fluorescence and lesion formation were determined as described in Frank et al. [6]. Measurement of the peroxide content expressed as H_2_O_2_ equivalents g^−1^ fresh weight (FW) was performed as described in Abuelsoud et al. [5].

### 4.4. RNA Isolation and Quantitative RT-PCR

A detailed protocol for RNA isolation can be found in Frank et al. [6]. cDNA synthesis and qRT-PCR analysis were performed as described in Cortleven et al. [65] using 500 ng of total RNA and a CFX96^TM^ Real-Time Touch System (Bio-Rad Laboratories GmbH; Feldkirchen, Germany). All primers used in this study are listed in Supplementary Table S4. Gene expression data were normalized against reference genes according to Vandesompele et al. [66]. *PROTEIN PHOSPHATASE 2A SUBUNIT A2* (*PP2AA2*, AT3G25800), *UBIQUITIN-CONJUGATING ENZYME 10* (*UBC10*, AT5G53300) and *METACASPASE 2D* (*MCP2D*, AT1G79340) served as reference genes.

### 4.5. RNA-Seq Data

A detailed description of sampling conditions, RNA-seq data acquisition and statistical analysis can be found in Cortleven et al. [33]. log_2_-fold values can be found in Supplementary Tables S1 and S2. Data presented in this study were prepared using Microsoft Excel^®^ 2016.

### 4.6. Determination of Auxin Concentration

For auxin measurements, 100 mg fresh weight of leaf tissue (leaves 8–12) per sample was collected and shock-frozen in liquid nitrogen under white light (time points during light exposure) or green safety light (time points during night). Analysis was carried out using high-performance liquid chromatography–electrospray tandem mass spectrometry as described in [67] using 10 mg tissue per biological replicate. Samples were extracted with 1 mL of 50 mM phosphate buffer (pH 7.0) containing 0.1% sodium diethyldithiocarbamate. [^13^C_6_]IAA, [^13^C_6_]oxIAA, [^13^C_6_]IAA-Glc, [^13^C_6_]oxIAA-Glc, [^13^C_6_]IAA-Asp and [^13^C_6_]IAA-Glu (5 pmol of each) were added as internal standards. A 200 µL portion of extract was acidified with 1M HCl to pH 2.7 and purified by in-tip micro solid-phase extraction (in-tip µSPE). After evaporation under reduced pressure, samples were analyzed using HPLC system 1260 Infinity II (Agilent Technologies, Santa Clara, CA, USA) equipped with Kinetex C18 column (50 mm × 2.1 mm, 1.7 µm; Phenomenex) and linked to 6495 Triple Quad detector (Agilent Technologies, Santa Clara, CA, USA). Auxin levels were quantified using stable-isotope-labeled internal standards as a reference.

### 4.7. Statistical Analysis

Statistical analysis of auxin measurements was performed with R version 4.1.0 and for all other data with SAS^®^Studio (SAS Compliance Department, NC, USA; https://odamid.oda.sas.com/SASStudio, last accessed on 5 March 2022). Homogeneity and homoscedasticity were tested by Shapiro–Wilk (*p* ≥ 0.95) and Levene tests (*p* ≥ 0.01) before ANOVA testing was performed followed by Tukey or Bonferroni (auxin measurements) post-hoc test. If assumptions were not met, transformations (*n*^−0.1^, *n*^0.01^, *n*^0.1^, *n*^0.2^, *n*^0.3^, *n*^0.5^, log, sqrt) were performed. Paired Wilcoxon test with Benjamini–Hochberg (BH) correction was performed if assumptions were still not met after transformation.

## Data Availability

The data presented in this study are available in the article and Supplementary Materials.

## Supplementary Materials

The following are available online at https://www.mdpi.com/article/10.3390/ijms23062936/s1.
