# Supplementary material for: The Photoperiod Stress Response in Arabidopsis thaliana Depends on Auxin Acting as an Antagonist to the Protectant Cytokinin
Source: Int J Mol Sci. 2022 Mar 8;23(6):2936. doi: 10.3390/ijms23062936 (PMC8955046; doi:10.3390/ijms23062936)
Supplement: Supplementary file 1 [file ijms-23-02936-s001.zip › ijms-1608110-supplementary.pdf]

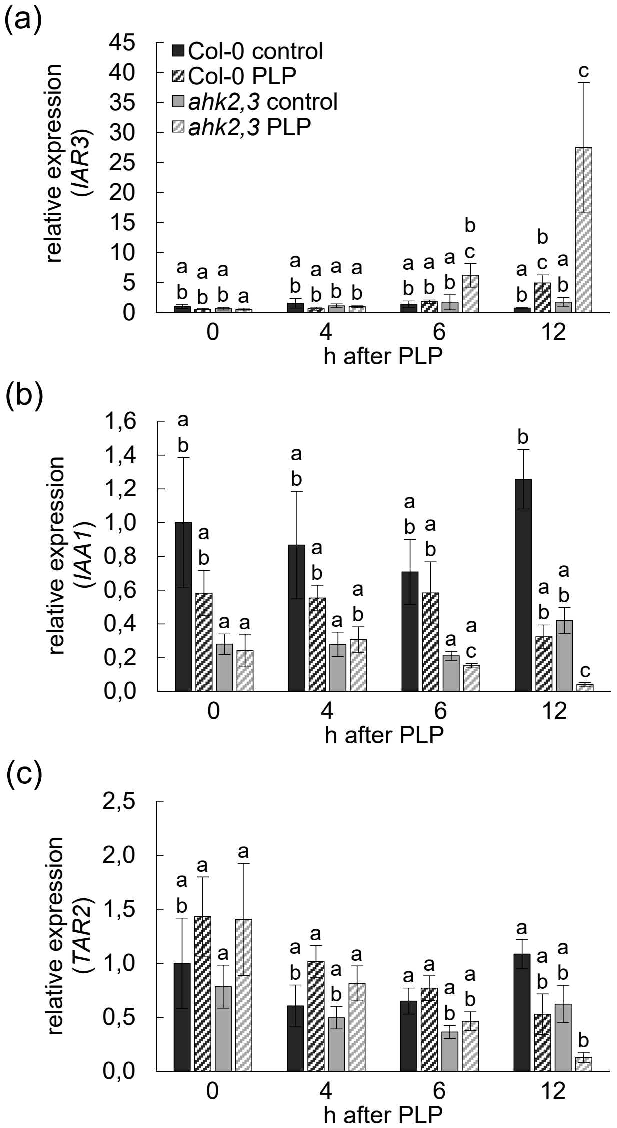

Supplemental Figure S1. qRT-PCR analysis of photoperiod stress-responsive auxin-related genes identified via RNA-seq. Transcript abundance of *IAR3* (a), *IAA1* (b) and *TAR2* (c) in wild type and *ahk2,3* 0, 4, 6 and 12 h after the PLP-treatment compared to respective control plants. The expression level of wild-type control at the end of the PLP treatment (0 h) was set to 1. Letters indicate significantly different statistical groups (one-way ANOVA; $p \leq 0.05$; $n \geq 3$). Error bars indicate SE. A schematic overview of the experimental setup can be found in Figure 1a.

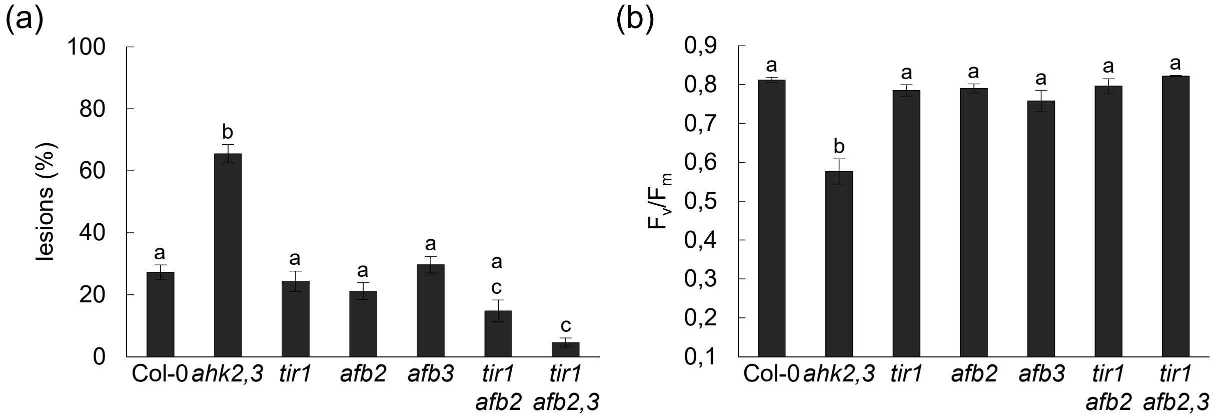

Supplemental Figure S2. Auxin receptors TIR1, AFB2 and AFB3 mediate the photoperiod stress sensitivity redundantly. (a) Lesion formation of leaves in five-week-old Col-0, *ahk2,3*, *tir1, afb2, afb3, tir1afb2* and *tir1afb2,3* plants the day after the PCD-inducing night (one-way ANOVA; $p \leq 0.05$; $n \geq 12$). (b) Efficiency of photosystem II (F_v/F_m) of leaves the day after the photoperiod stress treatment (Paired Wilcoxon test, FDR corrected via Benjamini-Hochberg test; $p \leq 0.05$; $n = 15$). Letters indicate significantly different statistical groups. Error bars indicate SE. Pictures of representative plants exposed to a 24-h prolongation of the light period are shown in Supplemental Figure S4c.

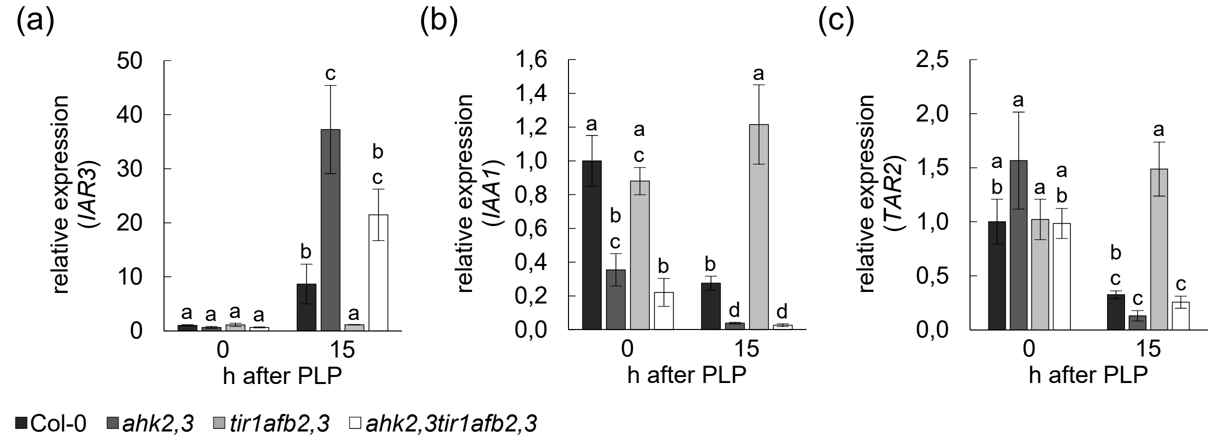

Supplemental Figure S3. Expression analysis of photoperiod stress-responsive auxin-related genes. Transcript abundance of *IAR3* (a), *IAA1* (b) and *TAR2* (c) 0 and 15 h after the PLP compared to respective control plants (same experimental setup as in Figure 5). The expression level of wild type at the end of the PLP treatment (0 h) was set to 1 (one-way ANOVA; p ≤ 0.05; n ≥ 3). Letters indicate significantly different statistical groups.

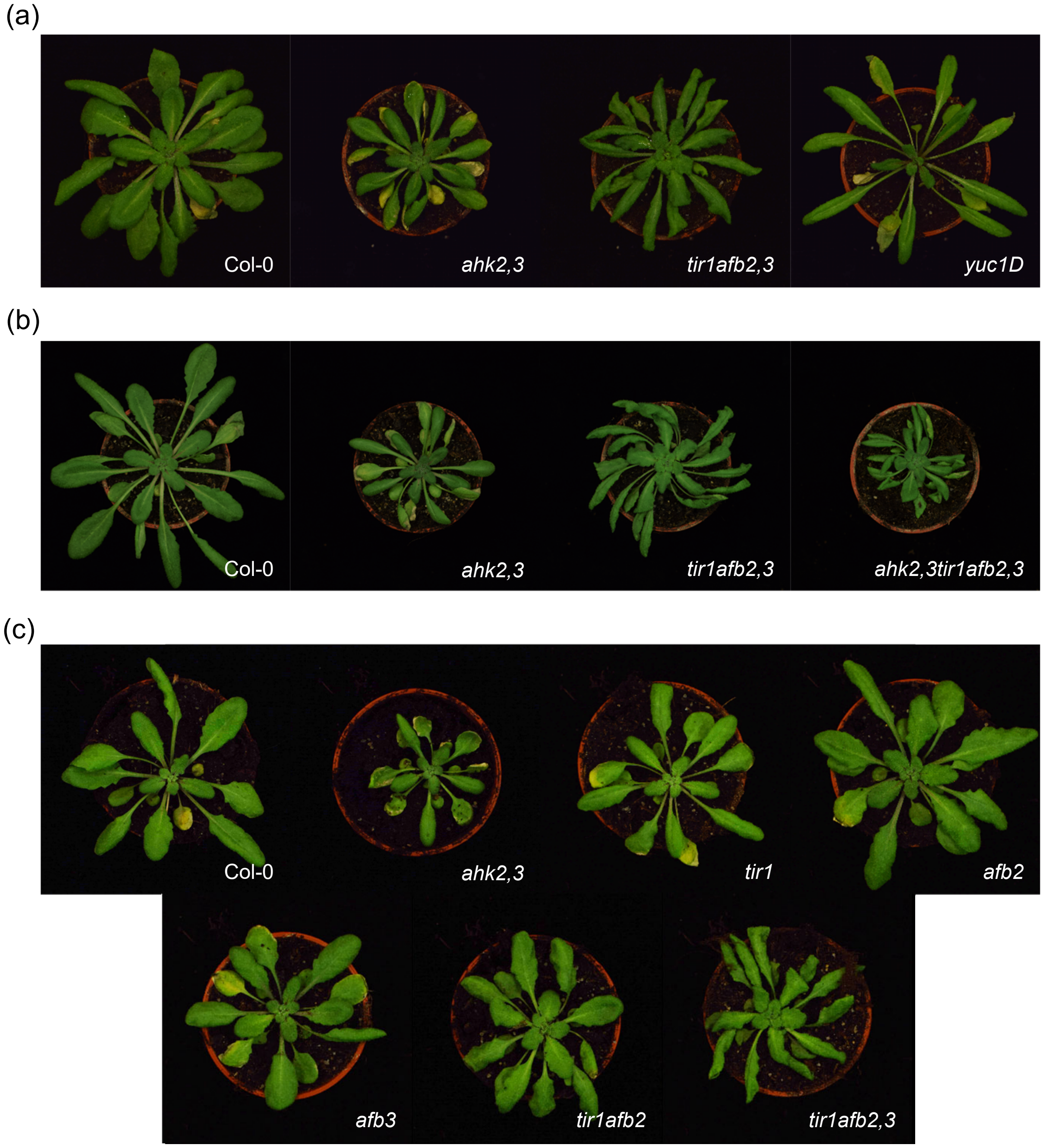

Supplemental Figure S4. Pictures of representative plants from experiments shown in this study. The plants were used for the experiments depicted in Figure 4 (a), Figure 5 (b) and Supplemental Figure S2 (c).

Supplemental Table S1. Overview over $\log_2$-fold changes of transcript abundance corresponding to plots depicted in Figure 1. Bold numbers indicate a significant difference between compared genotypes/conditions ($p \leq 0.05$; $n = 3$). Data have been extracted from [33]. PLP, prolonged light period.

	ahk2,3 control vs. WT control				WT PLP vs. WT control				*ahk2,3* PLP vs. *ahk2,3* control				*ahk2,3* PLP vs. WT PLP			
	0	4	6	12	0	4	6	12	0	4	6	12	0	4	6	12
TAA1	**-1.17**	**-1.19**	**-1.89**	**-2.32**	-0.53	-0.69	**-1.51**	-0.85	-1.05	**-1.63**	-0.84	-5.00	**-1.68**	**-2.13**	-1.22	**-6.47**
TAR1	-0.02	0.02	0.03	0.89	0.09	0.12	0.30	2.87	0.08	1.20	2.44	3.65	-0.03	1.10	2.17	1.68
TAR2	0.20	-0.49	-0.52	-0.45	0.98	0.86	0.68	-1.09	1.01	0.84	-0.16	**-3.80**	0.23	-0.50	-1.36	**-3.15**
YUC1	-0.02	0.02	-0.94	-0.07	0.09	-0.84	-0.66	0.02	0.08	-0.72	2.30	1.56	-0.03	0.14	2.02	1.47
YUC2	-1.53	-1.37	-0.71	-0.81	-0.43	-0.06	0.29	-0.42	0.98	0.76	-1.51	-5.11	-0.12	-0.56	-2.51	-5.49
YUC3	1.82	0.94	2.46	-0.07	0.09	-0.84	0.30	-0.94	-1.77	-1.64	-0.93	1.33	-0.03	0.14	1.23	2.20
YUC4	-0.73	-0.76	-1.65	-1.54	-1.61	-1.47	-2.71	-0.61	-1.33	-0.58	-0.20	-0.13	-0.45	0.13	0.86	-1.06
YUC5	-0.02	-0.56	-0.87	1.28	2.99	0.53	1.64	0.04	2.30	3.89	5.16	-2.62	-0.72	2.79	2.65	-1.38
YUC6	0.32	0.22	0.14	0.45	-0.51	-0.73	**-2.23**	**-1.43**	-0.40	**-1.73**	**-3.83**	**-3.99**	0.42	-0.77	-1.46	-2.11
YUC7	-0.02	0.98	-0.94	1.79	2.53	2.03	0.30	0.02	0.08	0.24	1.50	0.52	-2.47	-0.81	0.26	2.29
YUC8	-2.08	-2.24	-1.68	-1.19	-0.86	0.64	1.10	2.18	0.32	2.18	2.12	**2.84**	-0.89	-0.69	-0.66	-0.52
YUC9	-2.54	-0.51	-0.23	-0.63	-1.31	0.47	1.10	1.30	0.20	1.99	-0.69	-2.99	-1.03	1.01	-2.02	**-4.92**
YUC10	-0.02	0.02	-0.94	-0.07	0.09	0.12	-0.66	0.98	0.08	0.24	0.54	1.56	-0.03	0.14	0.26	0.51
YUC11	-0.02	0.02	0.03	-0.07	0.09	1.08	0.30	0.98	0.08	0.24	0.54	1.56	-0.03	-0.82	0.26	0.51
DAO1	-0.10	0.00	-0.27	-0.05	0.15	-0.03	0.10	0.76	0.16	-0.44	0.94	**2.13**	-0.09	-0.42	0.56	**1.32**
DAO2	-0.02	-2.14	0.03	-0.07	0.58	-1.10	1.82	0.02	0.56	1.22	1.41	4.91	-0.04	0.17	-0.39	4.82
IAR3	0.00	-0.52	-0.35	0.92	0.18	-0.05	1.45	**3.52**	0.16	1.41	**4.00**	**4.83**	-0.02	0.94	**2.21**	**2.23**
ILR1	-0.09	-0.03	0.04	-0.13	-0.12	-0.35	-0.38	0.79	-0.19	-0.26	0.77	**2.96**	-0.16	0.06	1.20	**2.04**
ILL1	0.01	0.07	0.06	-0.06	-0.03	-0.21	-0.46	**-0.59**	-0.06	-0.39	-0.69	**-1.70**	-0.01	-0.11	-0.17	**-1.18**
ILL2	-0.28	0.12	0.13	-0.10	-0.07	0.06	-0.03	-0.06	0.21	-0.06	-0.56	**-1.01**	0.00	0.00	-0.39	**-1.05**
GH3.1	1.81	0.98	-0.94	0.89	2.53	1.08	1.80	2.91	1.69	0.24	3.40	2.43	0.97	0.14	0.66	0.41
GH3.2	-3.35	-0.27	0.51	3.37	-0.77	2.28	**5.08**	**9.02**	2.52	3.38	**6.11**	**6.06**	-0.07	0.83	1.54	0.41
GH3.3	-2.17	-1.64	-1.86	-1.82	0.12	1.00	1.67	**6.17**	1.82	**3.72**	**3.28**	**5.53**	-0.47	1.09	-0.26	-2.46
GH3.4	-0.99	0.02	0.03	-0.07	-0.87	0.12	0.30	0.98	1.04	0.24	0.54	2.47	0.93	0.14	0.26	1.42
GH3.5	-0.39	0.40	1.02	0.76	-1.06	0.28	2.22	**5.42**	1.28	1.43	1.43	2.24	1.95	1.55	0.22	-2.42
GH3.6	-0.30	-1.40	-0.01	-0.36	0.60	0.46	1.38	1.14	0.15	0.84	1.86	**2.57**	-0.75	-1.02	0.47	1.07
GH3.9	-0.10	-0.54	0.42	-0.09	0.80	0.96	0.88	-0.07	1.44	1.56	-0.46	-1.92	0.54	0.06	-0.91	-1.94
GH3.17	1.12	0.84	1.60	1.18	0.40	0.49	-0.64	-0.03	0.32	-0.74	**-2.48**	-1.59	1.04	-0.39	-0.24	-0.37

Supplemental Table S2. Overview over log_2-fold changes of transcript abundance corresponding to plots depicted in Figure 2. Bold numbers indicate a significant difference between compared genotypes/conditions ($p \leq 0.05$; $n = 3$). Data have been extracted from [33]. PLP, prolonged light period.

	ahk2,3 control vs. WT control				WT PLP vs. WT control				*ahk2,3* PLP vs. *ahk2,3* control				*ahk2,3* PLP vs. WT PLP			
	0	4	6	12	0	4	6	12	0	4	6	12	0	4	6	12
TIR1	0.16	0.27	0.47	0.00	0.36	-0.34	-0.56	-0.98	0.42	**-1.28**	**-2.01**	**-2.84**	0.22	-0.66	**-0.98**	**-1.86**
AFB1	-0.09	0.08	0.45	0.33	-0.02	0.40	-0.09	0.08	0.53	-0.17	-1.60	**-2.63**	0.46	-0.48	-1.06	**-2.38**
AFB2	0.04	0.27	0.17	0.19	-0.38	-0.14	-0.24	-0.12	-0.14	-0.48	-0.34	0.14	0.28	-0.07	0.07	0.45
AFB3	0.20	0.14	0.01	-0.31	0.44	-0.14	-0.60	-0.51	0.36	**-0.93**	**-1.28**	**-1.54**	0.12	-0.65	-0.67	**-1.33**
AFB4	0.00	0.10	0.05	0.02	0.03	0.44	0.74	0.19	0.22	0.71	0.79	-0.01	0.19	0.37	0.09	-0.19
AFB5	0.17	0.16	0.13	-0.07	0.10	-0.46	-1.00	-1.17	0.05	**-1.13**	**-2.07**	**-2.80**	0.12	-0.51	**-0.94**	**-1.70**
IAA1	-0.99	-0.79	-0.70	-0.87	0.46	0.48	0.39	0.06	0.28	0.03	**-2.04**	**-2.17**	-1.17	-1.23	**-3.14**	**-3.09**
IAA2	-0.63	-0.20	0.22	0.19	0.38	0.92	1.58	1.09	0.82	**2.25**	**1.89**	0.86	-0.19	**1.13**	0.53	-0.04
IAA3	0.08	-0.62	-0.30	-0.26	-0.46	-0.13	-0.72	-0.71	-0.71	-0.13	**-1.76**	**-3.26**	-0.17	-0.62	-1.34	**-2.82**
IAA4	-0.16	-0.19	0.04	-0.06	0.05	-0.08	-0.67	-0.98	0.27	-1.22	**-2.94**	**-4.30**	0.06	-1.34	**-2.23**	**-3.37**
IAA5	-2.17	-2.93	-1.53	-2.05	0.46	0.54	0.20	-0.31	0.87	1.31	-2.29	-4.72	-1.76	-2.16	-4.01	**-6.46**
IAA6	-0.05	-0.74	-0.44	-0.39	0.41	0.08	-0.23	0.01	-0.31	0.30	-1.86	-3.12	-0.77	-0.52	-2.07	-3.51
IAA7	NA	NA	NA	NA	NA	NA	NA	NA	NA	NA	NA	NA	NA	NA	NA	NA
IAA8	0.05	0.06	0.06	-0.25	0.13	0.01	-0.01	**-1.01**	0.09	-0.35	**-0.75**	**-2.78**	0.01	-0.30	**-0.67**	**-2.02**
IAA9	-0.07	-0.05	0.15	0.27	-0.09	**0.45**	0.23	0.15	-0.07	**0.65**	-0.07	**-0.65**	-0.05	0.15	-0.15	**-0.52**
IAA10	-0.05	0.35	0.19	1.03	-0.11	1.27	**1.85**	**2.29**	0.09	**1.81**	**3.56**	**3.31**	0.15	0.89	**1.89**	**2.05**
IAA11	0.09	0.03	0.13	-0.11	-0.07	-0.06	-0.29	-0.45	-0.05	-0.38	**-1.14**	**-2.14**	0.11	-0.30	-0.71	**-1.80**
IAA12	0.19	0.39	0.17	-0.01	0.31	0.27	-0.60	**-1.40**	0.02	-0.65	**-2.04**	**-3.96**	-0.10	-0.53	**-1.28**	**-2.57**
IAA13	-0.20	0.09	0.15	0.22	-0.28	0.05	-0.73	-0.40	-0.04	-0.31	**-1.68**	**-1.33**	0.05	-0.26	-0.81	-0.71
IAA14	-1.00	-0.03	-0.06	-0.32	0.08	0.19	-0.92	-0.04	-0.23	**-1.87**	**-2.69**	**-1.99**	-1.32	**-2.10**	**-1.83**	**-2.26**
IAA15	-1.46	2.47	0.99	-1.89	-1.35	1.98	1.26	0.64	-0.88	0.95	-0.42	1.56	-0.99	1.44	-0.70	-0.97
IAA16	-0.39	0.01	0.17	-0.03	-0.52	-0.25	-0.07	0.55	0.04	-0.64	0.56	**1.29**	0.18	-0.39	0.80	0.72
IAA17	-0.47	-1.06	-0.36	-0.42	**1.85**	0.50	0.16	-1.09	1.34	0.72	-0.82	**-3.34**	-0.97	-0.85	-1.34	**-2.66**
IAA18	0.30	0.10	0.08	-0.02	-0.07	-0.50	-0.53	0.25	-0.14	-0.58	0.03	**1.39**	0.24	0.02	0.64	**1.12**
IAA19	-1.40	-1.51	-0.75	-0.92	0.14	1.38	**1.85**	**1.65**	0.75	**3.13**	**1.77**	-1.10	-0.78	0.25	-0.83	**-3.68**
IAA26	0.02	0.12	0.04	0.03	-0.07	0.03	-0.38	-0.08	0.08	-0.26	0.05	0.15	0.17	-0.17	0.47	0.26
IAA27	0.09	-0.51	0.61	0.33	-0.38	-0.52	-1.52	-1.50	-0.73	0.21	-1.74	**-3.64**	-0.26	0.23	0.39	-1.81
IAA28	-0.18	0.21	0.68	0.84	-0.63	-0.11	-0.32	-0.37	-0.29	0.54	0.28	-0.24	0.15	0.86	**1.27**	0.97
IAA29	**-2.66**	-1.26	-0.71	-1.62	0.29	1.68	1.90	1.30	1.83	1.74	-0.41	**-3.94**	-1.12	-1.20	**-3.03**	**-6.86**
ARF5	0.62	0.28	0.38	0.06	-0.34	-0.52	**-1.65**	-0.70	-0.71	-0.99	-1.06	0.28	0.25	-0.18	0.97	1.04
ARF6	0.21	-0.13	0.21	0.05	-0.28	-0.39	**-0.68**	-0.25	-0.04	-0.54	**-0.78**	-0.19	0.45	-0.28	0.10	0.11
ARF7	0.08	0.33	0.19	0.33	-0.17	0.48	**0.70**	**0.61**	0.08	**0.56**	**0.88**	**1.24**	0.33	0.41	0.38	**0.96**
ARF8	0.27	0.12	0.35	0.13	0.00	-0.31	-0.90	-1.13	0.04	-0.75	**-1.87**	**-3.04**	0.31	-0.31	-0.61	**-1.78**
ARF1	0.08	0.11	-0.02	-0.17	**-0.49**	-0.35	**-0.50**	-0.43	-0.45	**-0.49**	-0.26	-0.43	0.12	-0.03	0.22	-0.17
ARF2	-0.16	0.12	0.00	-0.03	**-0.55**	-0.09	0.28	0.29	-0.43	0.00	**0.78**	0.44	-0.04	0.22	**0.50**	0.13
ARF3	-0.12	-0.13	0.02	-0.34	-0.25	-0.62	**-1.01**	**-1.33**	-0.16	**-1.16**	**-2.22**	**-3.23**	-0.02	-0.66	**-1.19**	**-2.24**
ARF4	0.25	0.27	0.41	0.32	-0.46	-0.35	**-0.82**	**-1.11**	-0.39	-0.68	**-1.90**	**-3.63**	0.31	-0.06	-0.67	**-2.20**
ARF9	-0.23	0.08	0.00	-0.19	-0.05	-0.13	**-0.51**	-0.25	0.04	**-0.77**	**-1.18**	**-0.92**	-0.14	**-0.55**	**-0.67**	**-0.85**
ARF10	0.20	0.25	0.35	0.35	0.43	-0.43	**-0.76**	-0.54	0.31	**-0.84**	**-1.52**	**-1.71**	0.07	-0.16	-0.41	**-0.83**
ARF11	-0.27	-0.31	-0.25	-0.43	-0.51	-0.34	-0.34	-0.25	-0.41	-0.49	-0.12	-0.18	-0.18	-0.46	-0.04	-0.36
ARF12	-0.02	0.98	1.93	0.89	0.09	0.12	0.30	0.02	-0.88	-0.72	-1.36	-0.37	-0.99	0.14	0.26	0.51
ARF13	-0.02	-0.94	0.99	-0.07	-0.87	-0.84	0.30	0.02	-0.88	0.24	-0.42	0.60	-0.03	0.14	0.26	0.51
ARF14	-0.02	-0.94	1.88	-0.07	0.09	-0.84	2.13	0.98	0.08	0.24	-0.35	0.60	-0.03	0.14	-0.60	-0.45
ARF15	-0.99	0.02	0.03	-0.07	-0.87	0.12	0.30	0.02	0.08	1.20	1.50	1.56	-0.03	1.10	1.23	1.47
ARF16	-0.02	-0.15	-0.04	-0.19	0.06	-0.33	-0.36	**-0.81**	-0.21	**-0.62**	**-0.92**	**-1.04**	-0.29	**-0.44**	**-0.60**	-0.42
ARF17	-0.16	-0.11	-0.18	-0.13	0.08	-0.05	-0.30	-0.06	0.20	-0.43	-0.41	-0.55	-0.04	-0.48	-0.29	**-0.62**
ARF18	0.36	0.52	0.35	0.28	-0.20	-0.09	-0.10	-0.41	-0.09	-0.21	-0.11	**-2.03**	0.46	0.41	0.33	**-1.34**
ARF20	-0.02	0.02	0.99	-0.07	-0.87	-0.84	0.30	0.98	-0.88	-0.72	-0.42	0.60	-0.03	0.14	0.26	-0.45
ARF21	-0.02	-0.89	0.03	-0.07	1.05	-1.75	0.30	1.85	0.08	-0.72	2.39	0.60	-0.99	0.14	2.11	-1.32
ARF22	-0.99	-1.46	1.89	1.77	0.09	-1.36	2.74	1.86	1.04	1.11	-0.36	1.65	-0.03	1.01	-1.22	1.56

Supplemental Table S3. Auxin concentrations (pmol g^{-1} FW (mean ± SD)) corresponding to the plots depicted in Figure 3. tp, time point. PLP, prolonged light period.

Condition	tp	IAA	oxIAA	IAA-Asp	IAA-Glu	IAA-Glc	oxIAA-Glc
Col-0 control	1	16.9 ± 1.6	217.8 ± 26.5	2.1 ± 0.6	1.9 ± 0.7	75.7 ± 28.3	639.7 ± 84.4
ahk2,3 control	1	9.4 ± 0.7	118.2 ± 12.5	5.9 ± 0.6	2.9 ± 0.7	50.0 ± 10.6	356.5 ± 27.9
Col-0 PLP	1	21.3 ± 2.5	231.5 ± 26.7	3.3 ± 1.3	4.2 ± 1.5	121.9 ± 18.7	724.1 ± 72.7
ahk2,3 PLP	1	13.6 ± 0.9	114.7 ± 6.9	4.5 ± 1.1	3.2 ± 1.3	109.3 ± 18.5	353.3 ± 13.4
Col-0 control	2	18.7 ± 2.2	240.2 ± 20.1	1.7 ± 0.7	1.2 ± 0.3	56.0 ± 18.8	718.3 ± 68.4
ahk2,3 control	2	7.1 ± 1.4	114.8 ± 14.4	4.3 ± 1.2	1.6 ± 0.7	45.8 ± 4.0	336.8 ± 50.3
Col-0 PLP	2	39.1 ± 10.5	284.3 ± 24.3	7.5 ± 2.9	1.4 ± 0.4	126.0 ± 27.7	859.4 ± 69.9
ahk2,3 PLP	2	33.2 ± 3.1	122.4 ± 11.9	6.6 ± 0.8	1.0 ± 0.3	101.7 ± 13.4	344.0 ± 41.4
Col-0 control	3	16.5 ± 1.3	240.3 ± 17.8	1.7 ± 0.7	0.6 ± 0.2	83.2 ± 9.5	720.2 ± 54.6
ahk2,3 control	3	6.8 ± 0.7	110.7 ± 10.5	4.1 ± 0.5	1.3 ± 0.3	62.4 ± 16.2	326.3 ± 35.4
Col-0 PLP	3	17.7 ± 2.8	318.5 ± 29.9	9.4 ± 2.3	1.8 ± 0.7	151.5 ± 32.2	972.6 ± 59.6
ahk2,3 PLP	3	17.5 ± 2.1	189.6 ± 14.9	17.1 ± 2.4	1.1 ± 0.2	474.2 ± 94.9	562.1 ± 62.8

Supplemental Table S4. Sequences of primers used for qRT-PCR.

Gene	AGI number	Forward Primer	Reverse Primer	Source
BAP1	AT3G61190	CCAGAGATTACGGCGCGTGTT	TACAGACCCCAAACCGGAACTCC	Nitschke *et al*., 2016
CAB2	AT1G29920	AGAGGCCGAGGACTTGCTTTAC	GCCAATCTTCCGTTCTTGAGC	Nitschke *et al*., 2016
ZAT12	AT5G59820	CGCTTTGTCGTCTGGATTG	AGCAGCCCCACTCTCGTT	Nitschke *et al*., 2016
MCP2D	AT1G79340	AACCCGCTATGCAGACACACG	CAGTTGGTTTCCCCGCTGGA	Nitschke *et al*., 2016
PP2AA2	AT3G25800	CCATTAGATCTTGTCTCTCTGCT	GACAAAACCCGTACCGAG	Nitschke *et al*., 2016
UBC10	AT5G53300	CCATGGGCTAAATGGAAA	TTCATTTGGTCCTGTCTTCAG	Nitschke *et al*., 2016
IAA1	AT4G14560	CGGAGCTCCATATCTCCGTAAG	CATCACCGACCAACATCCAATC	this work
IAR3	AT1G51760	TTCCGCAGCATACCATAGATCC	CTTGCCTTGTGATAACCTGCTC	this work
TAR2	AT4G24670	ATGCAGGGATACGGATTGGATG	CCGTTACATTCCCACAACTGTC	this work
